# Diabolical points in coupled active cavities with quantum emitters

**DOI:** 10.1038/s41377-020-0244-9

**Published:** 2020-01-13

**Authors:** Jingnan Yang, Chenjiang Qian, Xin Xie, Kai Peng, Shiyao Wu, Feilong Song, Sibai Sun, Jianchen Dang, Yang Yu, Shushu Shi, Jiongji He, Matthew J. Steer, Iain G. Thayne, Bei-Bei Li, Fang Bo, Yun-Feng Xiao, Zhanchun Zuo, Kuijuan Jin, Changzhi Gu, Xiulai Xu

**Affiliations:** 1 Beijing National Laboratory for Condensed Matter Physics, Institute of Physics, Chinese Academy of Sciences, Beijing, 100190 China; 2 CAS Center for Excellence in Topological Quantum Computation and School of Physical Sciences, University of Chinese Academy of Sciences, Beijing, 100049 China; 3 School of Engineering, University of Glasgow, Glasgow, G12 8LT UK; 4 The MOE Key Laboratory of Weak Light Nonlinear Photonics, TEDA Applied Physics Institute and School of Physics, Nankai University, Tianjin, 300457 China; 5 State Key Laboratory for Mesoscopic Physics and Collaborative Innovation Center of Quantum Matter, School of Physics, Peking University, Beijing, China; 6 Songshan Lake Materials Laboratory, Dongguan, 523808 Guangdong China

**Keywords:** Micro-optics, Microresonators

## Abstract

In single microdisks, embedded active emitters intrinsically affect the cavity modes of the microdisks, resulting in trivial symmetric backscattering and low controllability. Here we demonstrate macroscopic control of the backscattering direction by optimizing the cavity size. The signature of the positive and negative backscattering directions in each single microdisk is confirmed with two strongly coupled microdisks. Furthermore, diabolical points are achieved at the resonance of the two microdisks, which agrees well with theoretical calculations considering the backscattering directions. Diabolical points in active optical structures pave the way for an implementation of quantum information processing with geometric phase in quantum photonic networks.

## Diabolically good control over quantum emitters

A system of tiny coupled disks developed by researchers in China and Scotland could provide control over the emission of individual photons for quantum computing. Single-photon emitters are required for passing information in quantum photonic networks, but it is very difficult to control the direction that photons are emitted or to stop neighboring emitters from interfering. Xiulai Xu at the Chinese Academy of Sciences and co-workers fabricated pairs of 1-micrometer-radius disks, surrounded by even smaller particles called quantum dots. By exciting the quantum dots with a laser, the researchers set up so-called diabolical points in the coupled microdisks. These points allow control over the backscattering of light, as a function of the distance between disks. The study not only provides a new platform for quantum information processing but could also enable controllable directional lasers.

## Introduction

Diabolical points (DPs) and exceptional points (EPs) describe degeneracies of systems depending on parameters^1,2^. EPs refer to degeneracies of non-Hermitian systems with coalescent eigenstates, which are quite popular in systems with gain and loss such as parity-time-symmetric systems^3–5^. DPs indicate the degeneracy of a Hermitian system with twofold orthogonal eigenstates. Compared to EPs with gain and loss, DPs have more practical feasibility, provide a geometric phase with a controlled phase shift, and introduce new approaches to the study of topological or quantum DP behaviors^6–11^. Thus photons in photonic structures at DPs have potential applications in quantum information and quantum computation^12–15^. Meanwhile, active emitters in photonic structures are essential for a coherent electron–photon interface to implement quantum information processing in a quantum photonic network^16–20^. However, the DPs or EPs of backscattering in optics can be achieved in optical structures with a few individually controlled defects or scatterers^21–23^. In active cavities with multiple quantum emitters, the quantum emitters affect the cavity mode as scatterers themselves^24,25^. The random positions of multiple emitters make the system difficult to control. More importantly, multiple scatterers result in symmetric backscattering in a single microdisk^26,27^. Symmetric backscattering forbids a degeneracy with only trivial eigenstates; thus a coherent interface between electrons and photons at DPs is difficult to achieve.

Single microdisks have two-dimensional Hamiltonians based on clockwise (CW) and counterclockwise (CCW) modes^28^. Symmetric backscattering results in splitting of the eigenstates, corresponding to the absolute value of the backscattering coupling strength. Previous studies on active microdisks mainly focused on the splitting in the spectrum, and further investigations have been limited by low controllability^29–31^. In contrast, two strongly coupled microdisks have supermodes with four-dimensional Hamiltonians. The detuning between the microdisks can be controlled, and not only the absolute value but also the sign of the backscattering coupling strength can be investigated. This feature makes coupled cavities a good platform for studying the fundamental physics of backscattering in active microdisks.

Here we demonstrate Hermitian degeneracy at DPs in two coupled microdisks with embedded quantum dots (QDs). Despite the low controllability originating from the randomly positioned QDs, macroscopic control by the cavity size is achieved based on the competition between the backscattering from QDs and defects^31^. Then the sign of the backscattering coupling strength is investigated via the coupling between the cavities. A balanced competition is clearly demonstrated by the distributed backscattering coupling strength from negative to positive values. Furthermore, the balanced competition provides the basis for the observation of Hermitian degeneracy at DPs, which occurs when the backscattering coupling strengths in two microdisks are opposite. Our work demonstrates DPs in active optical structures. The coupled cavities pave the way for scaling up quantum information processing^32–34^, and the QDs can serve as quantum emitters if brought into resonance with the cavity modes. Therefore, our work provides a potential approach to integrate photons at DPs into a quantum network in the future.

## Results

### Concept and design

**Fig. 1:**
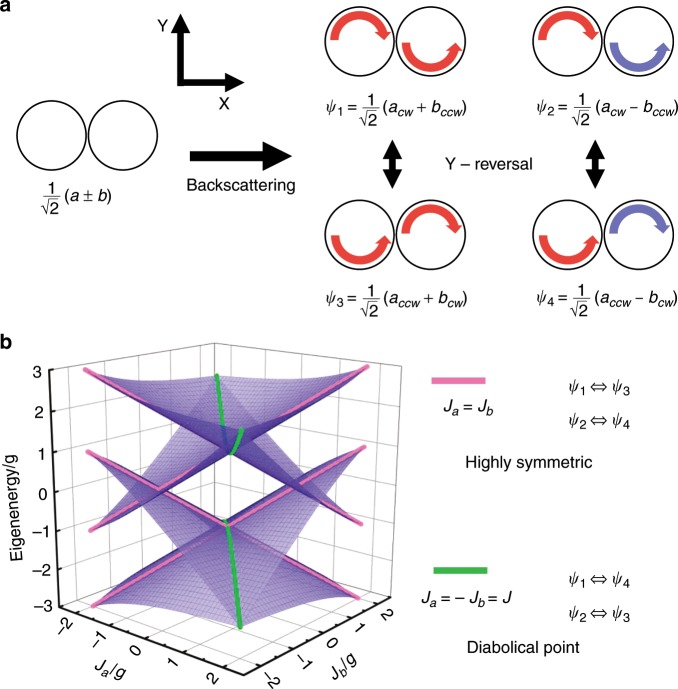
Schematics of two coupled cavities with backscattering and their eigenvalues. **a** Schematics of two pairs of reversal states with backscattering. The red arrows refer to +, while the blue arrows refer to −. **b** Four eigenvalues with different values of *J*_*a,b*_. The pink lines refer to results with *J*_*a*_ = *J*_*b*_. The green lines refer to results with *J*_*a*_ = −*J*_*b*_.

Two coupled microdisks (A and B) without backscattering have two eigenstates, as shown in Fig. 1a. For the perfect single microdisk A (B), the eigenstate is *a*(*b*) with the eigenvalue *ω*_*a*_(*ω*_*b*_). When two microdisks are coupled, there is a coupling strength *g* between them. When two microdisks are on resonance *ω*_*a*_ = *ω*_*b*_, the two eigenstates are $\psi = \left( {a \pm b} \right)/\sqrt 2$. For active microdisks with multiple scatterers, the degeneracy of the eigenstates of a single microdisk is lifted by backscattering. The CW and CCW modes of the single microdisk A (B) are *a*_cw,ccw_ (*b*_cw,ccw_), respectively. The backscattering in each cavity is symmetric between the CW and CCW modes, with strengths of *J*_*a*_ for microdisk A and *J*_*b*_ for microdisk B. The frequencies of the cavity modes *ω*_*a,b*_ and the backscattering *J*_*a,b*_ can contain an imaginary part corresponding to energy loss^21,25^. The coupling between the cavities is only allowed between states with the same propagation directions (*a*_cw_ and *b*_ccw_, *a*_ccw_ and *b*_cw_) with strength *g*^35^. Owing to the backscattering, the two pairs of originally degenerate reversal states *ψ*_1,3_ and *ψ*_2,4_ (Fig. 1a) now couple to each other, resulting in new eigenstates.

The Hamiltonian on resonance (*ω*_*a,b*_ is set to 0 for brevity) with the basis vector *ψ*_*i*_ is

$$\left( {\begin{array}{*{20}{c}} g & 0 & {(J_a + J_b)/2} & {(J_a - J_b)/2} \\ 0 & { - g} & {(J_a - J_b)/2} & {(J_a + J_b)/2} \\ {(J_a + J_b)/2} & {(J_a - J_b)/2} & g & 0 \\ {(J_a - J_b)/2} & {(J_a + J_b)/2} & 0 & { - g} \end{array}} \right)$$

where the order of the basis in the matrix is *ψ*_1_ to *ψ*_4_. Figure 1b shows the calculated eigenvalues with real backscattering coupling strengths. As shown in the Hamiltonian above, the internal coupling of the system is significantly affected by the sign of the backscattering coupling strength. When *J*_*a*_ = *J*_*b*_ (pink lines), the system is highly symmetric, and the coupling occurs between the reversal states, as shown in Fig. 1a. When *J*_*a*_ = −*J*_*b*_ (green lines), the coupling between the reversal states is destructive and only occurs between *ψ*_1_ and *ψ*_4_ or between *ψ*_2_ and *ψ*_3_. Eigenstates without reversal symmetry in the system with reversal symmetry indicate spontaneous symmetry breaking^36^. Furthermore, the system only has two eigenvalues, corresponding to Hermitian degeneracy at the DPs.

The degenerate eigenspace at the DPs is an important feature, providing the basis for quantum states with continuous phases. The eigenstates can also be expressed by phases $\theta _a,\;\phi _a,\;\theta _b,\;\phi _b,\;\phi _1,\;{\mathrm{and}}\;\phi _2$ as

$$S^\prime = \sin \phi _1{\mathrm{e}}^{i\phi _2/2}\left( {\sin \theta _a{\mathrm{e}}^{i\phi _a/2}a_{{\mathrm{cw}}} + \cos \theta _a{\mathrm{e}}^{ - i\phi _a/2}\;a_{{\mathrm{ccw}}}} \right) + \cos \phi _1{\mathrm{e}}^{ - i\phi _2/2}\left( {\sin \theta _b{\mathrm{e}}^{i\phi _b/2}b_{{\mathrm{ccw}}} + \cos \theta _b{\mathrm{e}}^{ - i\phi _b/2}\;b_{{\mathrm{cw}}}} \right)$$

**Fig. 2:**
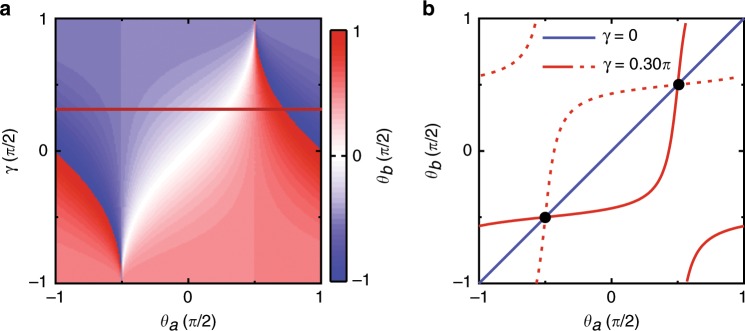
Correlation between phases of two microdisks at DP. **a** One eigenspace with different values of *γ*, *J*_*a*_ = −*J*_*b*_ = *J* and *ϕ*_*a*_ = *ϕ*_*b*_ = 0. This eigenspace refers to the upper green line in Fig. 1 **b**. **b** Eigenspaces and correlations between two microdisks. The black dots refer to trivial systems. The blue line refers to a DP without backscattering. The red lines refer to two DPs with backscattering for *γ* = 0.30 *π* with a phase shift. The solid red line corresponds to the red line in (a) and a point in the upper green line in Fig. 1 (**b**). The dashed red line corresponds to a point in the bottom green line in Fig. 1 (**b**).

In this form, the phase of the left microdisk is defined by the normalized amplitudes of *a*_cw_ and *a*_ccw_ with *θ*_*a*_ and *ϕ*_*a*_. The normalized amplitude of *a*_cw_ is ${\mathrm{sin}} \, \theta _a{\mathrm{e}}^{i\phi _a/2},$ and the normalized amplitude of *a*_ccw_ is ${\mathrm{cos}} \, \theta _a{\mathrm{e}}^{ - i\phi _a/2}$ ^37,38^. Similarly, the phase of the right microdisk is defined by *θ*_*b*_ and *ϕ*_*b*_, where the normalized amplitude of *b*_ccw_ is ${\mathrm{sin}} \, \theta _b{\mathrm{e}}^{i\phi _b/2}$ and the normalized amplitude of *b*_*cw*_ is ${\mathrm{cos}} \, \theta _b{\mathrm{e}}^{ - i\phi _b/2}$. The twofold degeneracy in the four-dimensional Hamiltonian results in two two-dimensional eigenspaces, and the reduced degrees of freedom result in correlation between the phases of the two microdisks. Figure 2a shows one eigenspace at the DP, and the correlation is $\tan \theta _b = \left( {\tan \theta _a - \sin \gamma } \right)/\left( {1 - \tan \theta _a\sin \gamma } \right)$, where tan*γ* = *J*/*g*. Figure 2b shows the advantage of DPs with the phase shift by a comparison between different cases. Without degeneracy, the system is trivial (nondegenerate) and only permitted at the black dots. The blue line refers to the eigenspace at the DP without backscattering, which is linear with no phase change between two microdisks. The solid (dashed) red line refers to a point in the upper (bottom) green line in Fig. 1b at the DP with backscattering. The nonlinear correlations result in a phase shift between two microdisks, which is potentially applicable to quantum networks. For example, if waveguides are coupled to the microdisks, the phases of the CW and CCW modes in the microdisks are related to the forward and backward signals in the waveguides. Thus the coupled microdisks at the DP can be used for the phase shift of a signal in two waveguides as a quantum node. Meanwhile, *γ*(*g*) can be controlled by the gap between the two microdisks but does not affect the DP (*J*_*a*_ = −*J*_*b*_), indicating more potential applications such as controllable directional lasers. More detailed calculations can be found in Supplementary Information.

### Macroscopic control to achieve DPs

**Fig. 3:**
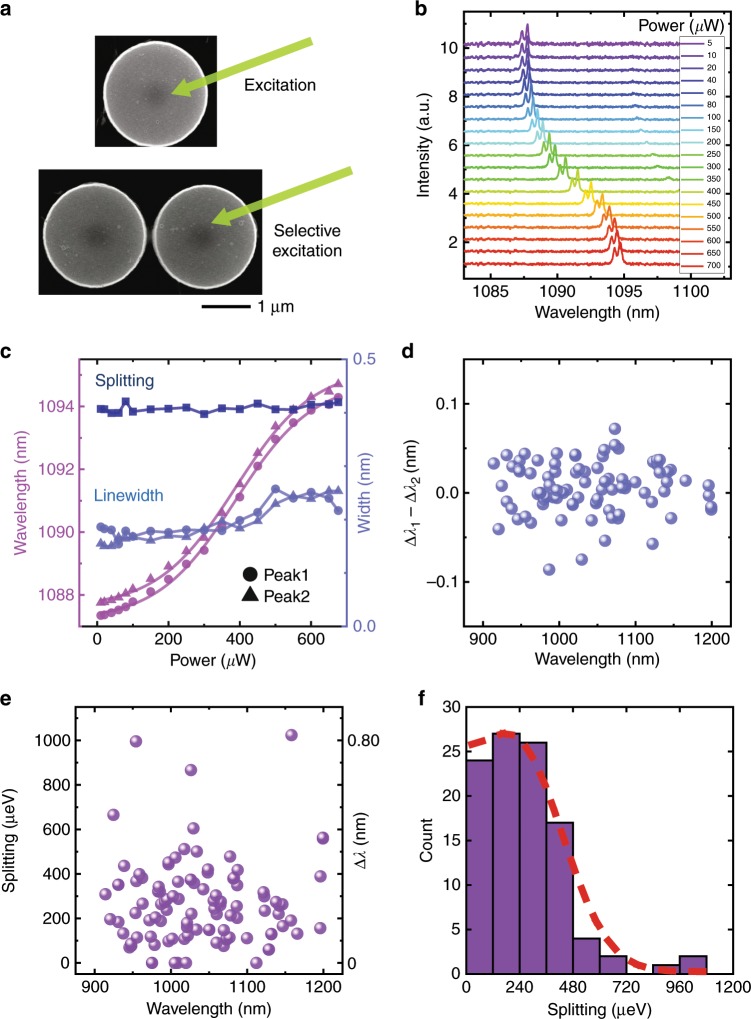
Characteristics of cavity modes with backscattering. **a** SEM images of a single microdisk and double microdisks. The excitation laser is labeled by the green arrow. **b** The redshift of the cavity modes with increasing excitation power. **c** The wavelengths, linewidths, and splitting between the two peaks extracted from Lorentzian multipeak fitting. **d** Statistics of the linewidth differences between the split modes. The resolution of the spectrometer is 0.1 nm. **e** Statistics of the splitting. A splitting of 1000 μeV corresponds to 0.80 nm at a wavelength of 1000 nm. **f** Distribution of the splitting and half-Gaussian fitting.

In an experiment, microdisks with a radius of 1 μm were fabricated on a 250-nm-thick GaAs slab. One layer of InAs QDs was grown in the center of the slab with a density of 30 μm^−2^. The gap between the microdisks was designed to range from 50 to 130 nm. Figure 3a shows scanning electron microscopic (SEM) images of the cavities. The QDs were excited by a laser with a wavelength of 532 nm at 4.2 K. Figure 3b shows two spectrally resolved peaks resulting from backscattering in a single microdisk. The cavity modes are redshifted by 7 nm by the thermo-optic effect with increasing excitation power^39^. Meanwhile, the splitting and peak linewidths are barely affected, as shown in Fig. 3c. Therefore, the detuning between the two microdisks can be controlled by a selective excitation.

For the two coupled microdisks, the DPs require *ω*_*a*_ = *ω*_*b*_ and *J*_*a*_ = −*J*_*b*_ for both the real and imaginary parts. In realistic active microdisks, the backscattering is difficult to precisely control. The approaches used to control backscattering in passive microdisks are invalid here due to the randomly positioned QDs. Instead, the devices are designed to improve the possibility of DPs. The possibility of equal imaginary parts of *ω*_*a,b*_ (linewidth) is improved by the identical design and fabrication of the two coupled microdisks. The linewidth difference between the two split modes, which is the imaginary part of *J*, is smaller than the resolution of our spectrometer, as shown in Fig. 3d. The linewidth difference (average value of 0.02 nm) is also much smaller than the mode splitting (average value of 0.24 nm as shown in Fig. 3e), which represents the real part of *J*. Thus the imaginary part of *J*_*a,b*_ is almost zero. The symmetric backscattering and the very small imaginary part can be attributed to an average effect of randomly positioned multiple scatterers, as discussed in Supplementary Information. Then the main challenge for the DPs is to control the system toward opposite real parts of the backscattering coupling strength *J*_*a*_ = −*J*_*b*_. To solve the problem of low controllability, we propose to use macroscopic control based on the competition between different types of scatterers.

The microdisk contains two types of scatterers. One type is the defects on the surface, and the other type is the embedded QDs^40,41^. Although the detailed distribution of defects and QDs is random, the main role of the two types of scatterers is related to the perimeter/area ratio determined by the microdisk size. Previous studies mainly focused on splitting in single microdisks, corresponding to the absolute value of the backscattering coupling strength^29–31^. Thus the competition between the two types of scatterers was only qualitatively described^31^. In contrast, the competition here is further investigated, including the sign of the backscattering coupling strength. The backscattering of the scatterers is related to the difference between the dielectric permittivities of the scatterers and the surrounding medium^21,25,29^. Defects serve as low-refractive-index scatterers with positive contributions to *J*, and conversely, QDs serve as high-refractive-index scatterers with negative contributions. Thus the sign of the backscattering coupling strength is affected by the dominant type of scatterers. When the competition is balanced, both positive and negative values of *J* can be predicted from the distribution, paving the way for DPs at *J*_*a*_ = −*J*_*b*_. Based on the results in previous work^31^ and the parameters of our devices, the microdisk radius is designed to be 1 μm for balanced competition. Figure 3e, f show the statistics of the splitting 2|*J*| with a nearly half-Gaussian distribution, corresponding to a Gaussian distribution of *J* with a mean value close to zero. This result demonstrates good balance in the competition. More design and fabrication details are shown in Supplementary Information.

**Fig. 4:**
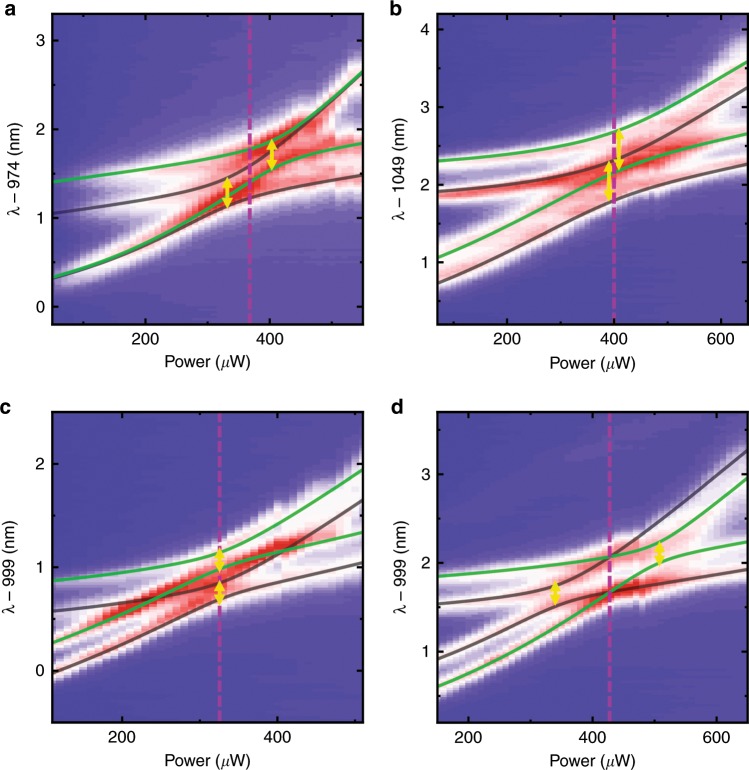
Excitation-power-dependent PL maps of the coupled cavities and the fitted results with different values of *J*_*a,b*_. The resonance *ω*_*a*_ = *ω*_*b*_ is marked by purple dashed lines. **a** *J*_*a*_ = 0 and *J*_*b*_ ≠ 0. **b**, **c** *J*_*a*_*J*_*b*_ > 0. **c** *J*_*a*_ = *J*_*b*_. **d** *J*_*a*_*J*_*b*_ < 0 and *J*_*a*_ = −*J*_*b*_. The DPs occur on resonance.

Excitation-power-dependent photoluminescence (PL) spectroscopy by a selective excitation was performed on various coupled microdisks. Figure 4 shows four typical PL mappings of supermodes as well as theoretical fits (solid lines). Two anti-crossings (yellow arrows) indicating strong couplings between the supermodes (one pair of green lines and another pair of gray lines) are observed in all measurements. The two and only two anti-crossings are the result of symmetric backscattering. More detailed discussions and additional data are shown in Supplementary Information. Figure 4a shows the case for *J*_*a*_ = 0 and *J*_*b*_ ≠ 0, which means that splitting in the first microdisk is not observed. Figure 4b, c shows the case for *J*_*a*_*J*_*b*_ > 0, which means that the backscattering coupling strengths in the two microdisks are both positive or both negative. In particular, Fig. 4c shows the case for *J*_*a*_ = *J*_*b*_, which results in two simultaneous anti-crossings on resonance (purple dashed line). Figure 4d shows the remarkable case with Hermitian degeneracy at the DPs, where *J*_*a*_ = −*J*_*b*_. Strong couplings occur between the different pairs of supermodes compared to the coupling behaviors of two pairs of supermodes in Fig. 4b, c, which is the key difference between the cases with *J*_*a*_*J*_*b*_ > 0 and *J*_*a*_*J*_*b*_ < 0. The different couplings refer to the significance of the sign of the backscattering coupling strength in coupled cavities, in contrast to previous work where only the absolute value is characterized by resolving the splitting in single cavities^29–31^.

## Discussion

The fitted results in Fig. 4d show a coupling strength of *g* = 145 μeV and *J*_*a*_ = −*J*_*b*_ = 200 μeV. The linewidths of all four peaks are approximately 0.20 nm. This means that the two cavities are brought into resonance, where *ω*_*a*_ = *ω*_*b*_ for both the real and imaginary parts. Meanwhile, the same linewidth of the two peaks from the single microdisks indicates that the imaginary parts of *J*_*a*_ and *J*_*b*_ are zero. Therefore, the Hermitian degeneracy and DPs on resonance (purple dashed line) are demonstrated, in good agreement with the theoretical result shown by the green lines in Fig. 1b. The eigenstates split by the backscattering in the single microdisks are degenerate because of the coupling between the two microdisks. The ratio of the backscattering coupling strength to the coupling strength between the microdisks is *J*/*g* = 1.38 = tan(0.30*π*). Thus the two Hermitian degeneracies correspond to two paths, as shown by the red lines in Fig. 2b. The achieved DPs demonstrate the potential of macroscopic control in active microdisks with multiple QDs. In addition, as fabrication technology and QD growth techniques improve^42,43^ and *g* is controlled by a tunable gap^44,45^, backscattering in coupled active cavities may also be precisely controlled in the future.

In summary, we have demonstrated DPs in two strongly coupled active microdisks. The coupling between the cavities reveals that the sign of the backscattering coupling strength is an important physical property. Macroscopic control of the backscattering is achieved based on a competition between defects and emitters, solving the problem of low controllability originating from randomly positioned scatterers. The competition is balanced by an optimized microdisk size and experimentally demonstrated, providing the basis for the successful observation of DPs. This work paves the way for DPs or EPs in optical structures with active emitters and thus has potential for applications in quantum photonic networks. In addition to individual quantum devices^46–49^, coupled cavities can also be designed with more exotic phenomena and applications.

## Materials and methods

### Growth of the sample with QDs

The sample for our device was grown by molecular beam epitaxy, which consists of a 250-nm-thick GaAs slab, a 1-μm-thick AlGaAs sacrificial layer, and a GaAs substrate. One layer of self-assembled QDs was grown at a low growth rate to achieve a low density and a large dot size in the middle of the GaAs slab. The QD density is approximately 30 μm^−2^. One ground state and at least two excited states could be observed from the PL spectrum of ensemble QDs. The wavelength of the ground state is approximately 1200 nm, and the wavelength of the first excited state is approximately 1120 nm.

### Microdisk fabrication

Microdisks were fabricated by employing electron beam lithography to pattern the resist, followed by dry etching using induced coupled plasma to form circular pillars. Then wet etching using hydrofluoric acid solutions was performed to dissolve the sacrificial layer and form a supporting pillar. The gaps between the two microdisks were designed to range from 50 to 130 nm.

### Optical measurement

The optical measurement was implemented with a conventional confocal microPL system. The device was mounted on a three-dimensional nanopositioner and cooled down to 4.2 K by exchanging helium gas with a helium bath. A solid-state laser with an emission wavelength at 532 nm was first used to selectively excite and heat one of the microdisks. The excited GaAs substrate then excites the wetting layer below the QDs, and the QDs are subsequently excited. Finally, all the cavity modes were excited by the QDs within their spectral range of emission. Owing to the random emission direction of the QDs, the QDs will not selectively excite CW or CCW modes. The PL spectra were collected by a linear array of InGaAs detectors dispersed through a spectrometer with a resolution of 0.1 nm.

## Supplementary information

Supplemental Material in PDF

## Data Availability

The data that support the plots within this paper and Supplementary Information are available from the corresponding author upon request.
