# Supplementary material for: Diabolical points in coupled active cavities with quantum emitters
Source: Light Sci Appl. 2020 Jan 13;9:6. doi: 10.1038/s41377-020-0244-9 (PMC6957493; doi:10.1038/s41377-020-0244-9)
Supplement: Supplementary file 1 — Supplemental Material in PDF [file 41377_2020_244_MOESM1_ESM.pdf]

Supplemental Information for Diabolical Points in Coupled Active Cavities with Quantum Emitters

Jingnan Yang,[1,2,*] Chenjiang Qian,[1,2,*] Xin Xie,[1,2] Kai Peng,[1,2] Shiyao Wu,[1,2] Feilong Song,[1,2] Sibai Sun,[1,2] Jianchen Dang,[1,2] Yang Yu,[1,2] Shushu Shi,[1,2] Jiongji He,[1,2] Matthew J. Steer,[3] Iain G. Thayne,[3] Bei-Bei Li,[1] Fang Bo,[4] Yun-Feng Xiao,[5] Zhanchun Zuo,[1,2] Kuijuan Jin,[1,2,6] Changzhi Gu,[1,2] and Xiulai Xu[1,2,6,†]

[1] *Beijing National Laboratory for Condensed Matter Physics, Institute of Physics, Chinese Academy of Sciences, Beijing 100190, China*

[2] *CAS Center for Excellence in Topological Quantum Computation and School of Physical Sciences, University of Chinese Academy of Sciences, Beijing 100049, China*

[3] *School of Engineering, University of Glasgow, Glasgow G12 8LT, U.K.*

[4] *The MOE Key Laboratory of Weak Light Nonlinear Photonics, TEDA Applied Physics Institute and School of Physics, Nankai University, Tianjin 300457, China*

[5] *State Key Laboratory for Mesoscopic Physics and Collaborative Innovation Center of Quantum Matter, School of Physics, Peking University, Beijing, China*

[6] *Songshan Lake Materials Laboratory, Dongguan, Guangdong 523808, China*

(Dated: December 10, 2019)

* Contributed equally to this work.

† xlxu@iphy.ac.cn

CONTENTS

I. Detailed Theory and Extended Data:

A. Eigenstates for Coupled Cavities

B. Experimental Evidence for Symmetric Backscattering

C. Eigenspace at Diabolical Points

II. Fabrication and Measurement:

A. Material and Sample Fabrication

B. Measurement Methods

III. Balanced Competition

References

I. DETAILED THEORY AND EXTENDED DATA:

A. Eigenstates for Coupled Cavities

For two coupled microcavities without backscattering, the Hamiltonian can be expressed as

$$H_0 = \begin{pmatrix} \omega_a & g \\ g & \omega_b \end{pmatrix}$$

where g is the coupling strength between the two single microdisks with eigenstates (eigenvalues) respectively annotated by a (ω_a) and b (ω_b). When the two microdisks are at resonance $\omega_a = \omega_b$, two eigenstates are $\psi = (a \pm b) / \sqrt{2}$. While in fact, each eigenstate is two-fold degenerate when the propagation direction is considered, as

$$\psi_1 = \frac{1}{\sqrt{2}}(a_{cw} + b_{ccw}), \psi_2 = \frac{1}{\sqrt{2}}(a_{cw} - b_{ccw}),$$

$$\psi_3 = \frac{1}{\sqrt{2}}(a_{ccw} + b_{cw}), \psi_4 = \frac{1}{\sqrt{2}}(a_{ccw} - b_{cw}),$$

where $a_{cw,ccw}$ $(b_{cw,ccw})$ are the CW and CCW modes in two single microdisks. When scatterers are introduced into microdisks, the two pairs of two-fold degenerate eigenstates are lifted by the backscattering of mode field from the scatterers [1].

In single microdisks with backscattering, the Hamiltonian can be expressed as

$$H_a = \begin{pmatrix} \omega_a & J_{a1} \\ J_{a2} & \omega_a \end{pmatrix}$$

where $J_{a1,a2}$ are the backscattering coupling strengths. For each one scatterer, $J_{a1,a2}$ are complex with different amplitude and angle. Thus, in single passive microdisks with a few scatterers, the Hamiltonian is asymmetric with non-trivial states, such as exceptional points can be achieved. While in active microdisks, there are multiple randomly positioned scatterers. The difference in the amplitude and angle for each scatterer is averaged to zero in the ensemble system. Then we can get the symmetric backscattering $J_{a1} = J_{a2} = J_a$ ($J_{b1} = J_{b2} = J_b$ in microdisk B) as mentioned in the paper. This corresponds well to previous works on active microdisks, where no results on non-trivial states have been reported. The symmetric backscattering in our work can also be demonstrated by the coupling between two microdisks as shown in the next paragraph.

Then for the two coupled active microdisks in the paper, the Hamiltonian is written with the basis a_{cw}, a_{ccw}, b_{cw} and b_{ccw} as

$$\begin{array}{c c} & \begin{array}{cccc} a_{cw} & a_{ccw} & b_{cw} & b_{ccw} \end{array} \\ \begin{array}{c} a_{cw} \\ a_{ccw} \\ b_{cw} \\ b_{ccw} \end{array} & \begin{pmatrix} \omega_a & J_a & 0 & g \\ J_a & \omega_a & g & 0 \\ 0 & g & \omega_b & J_b \\ g & 0 & J_b & \omega_b \end{pmatrix}. \end{array}$$

where the elements which are zero identify no coupling between modes due to the mismatch of propagation directions [2]. J_a (J_b) is the backscattering coupling strength between a_{cw} and a_{ccw} (b_{cw} and b_{ccw}). Due to the symmetric backscattering, the Hamiltonian is invariant under Y-reversal operation which swaps the CW and CCW modes of each microdisk ($a_{cw} \Leftrightarrow a_{ccw}$ and $b_{cw} \Leftrightarrow b_{ccw}$). The eigenvalues are

$$\Omega_{1,2} = \frac{\omega_a+\omega_b-J_a-J_b}{2} \pm \sqrt{g^2 + \frac{(\omega_a-\omega_b-J_a+J_b)^2}{4}},$$

$$\Omega_{3,4} = \frac{\omega_a+\omega_b+J_a+J_b}{2} \pm \sqrt{g^2 + \frac{(\omega_a-\omega_b+J_a-J_b)^2}{4}}.$$

It can be clearly observed that the eigenvalues are the solutions of two quadratic equations. If the detuning between ω_a and ω_b is controlled, there will be two anticrossings with two pairs of coupled modes in the spectra. $\Omega_{1,2}$ are one pair of coupled modes and $\Omega_{3,4}$ are the other pair of coupled modes. It is important to note that the two, and only two, anticrossings are a significant feature originating from the symmetric backscattering $J_{a1} = J_{a2}$ and $J_{b1} = J_{b2}$. In contrast, if the backscattering is asymmetric, the eigenvalue will be the solutions of a quartic equation, and quite complex supermodes along with more anticrossings would be predicted in the spectra.

B. Experimental Evidence for Symmetric Backscattering

The two, and only two, anticrossings correspond very well with all experimental data in our work, demonstrating the symmetric backscattering $J_{a1} = J_{a2}$ and $J_{b1} = J_{b2}$. In the experiment, the detuning between two microdisks is controlled by the selective excitation. The peak information is first extracted from PL spectra by the multi-Lorentz fitting. Then the thermo-optic effect, which means the bare cavity mode wavelength if there is no coupling between two cavities, is expressed by $\omega_a = \omega' + B \,/\, \left(1 + e^{(P-P_0)/C}\right)$ [3] as discussed later in Sec. IIB. Finally, the experimental results were fitted by the theoretical analysis in Sec. IA to get the parameters g and $J_{a,b}$ as shown by green and black lines in SFig. 1. As mentioned in the paper, the selective excitation only changes the detuning between two microdisks, and does not affect other parameters such as the backscattering coupling strength. SFigure 1 shows four additional excitation-power-dependent PL mappings. They are similar to the results in Fig. 4 in the paper, containing J_a and J_b in different cases. Among all the experimental results, including those not shown here, there are two and only two anticrossings as marked by the two pair of color lines (green and black). To clearly show the crossings and anticrossings, SFig. 2 shows the enlarged spectra of the result in Fig. 3(c) in the paper as a typical example. The crossings marked by blue arrows and anticrossings marked by red arrows can be clearly observed. Additionally, more complex

spectra with more anticrossings, which correspond to the asymmetric backscattering $J_{a1} \neq J_{a2}$ or $J_{b1} \neq J_{b2}$, have not been observed in our experiment.

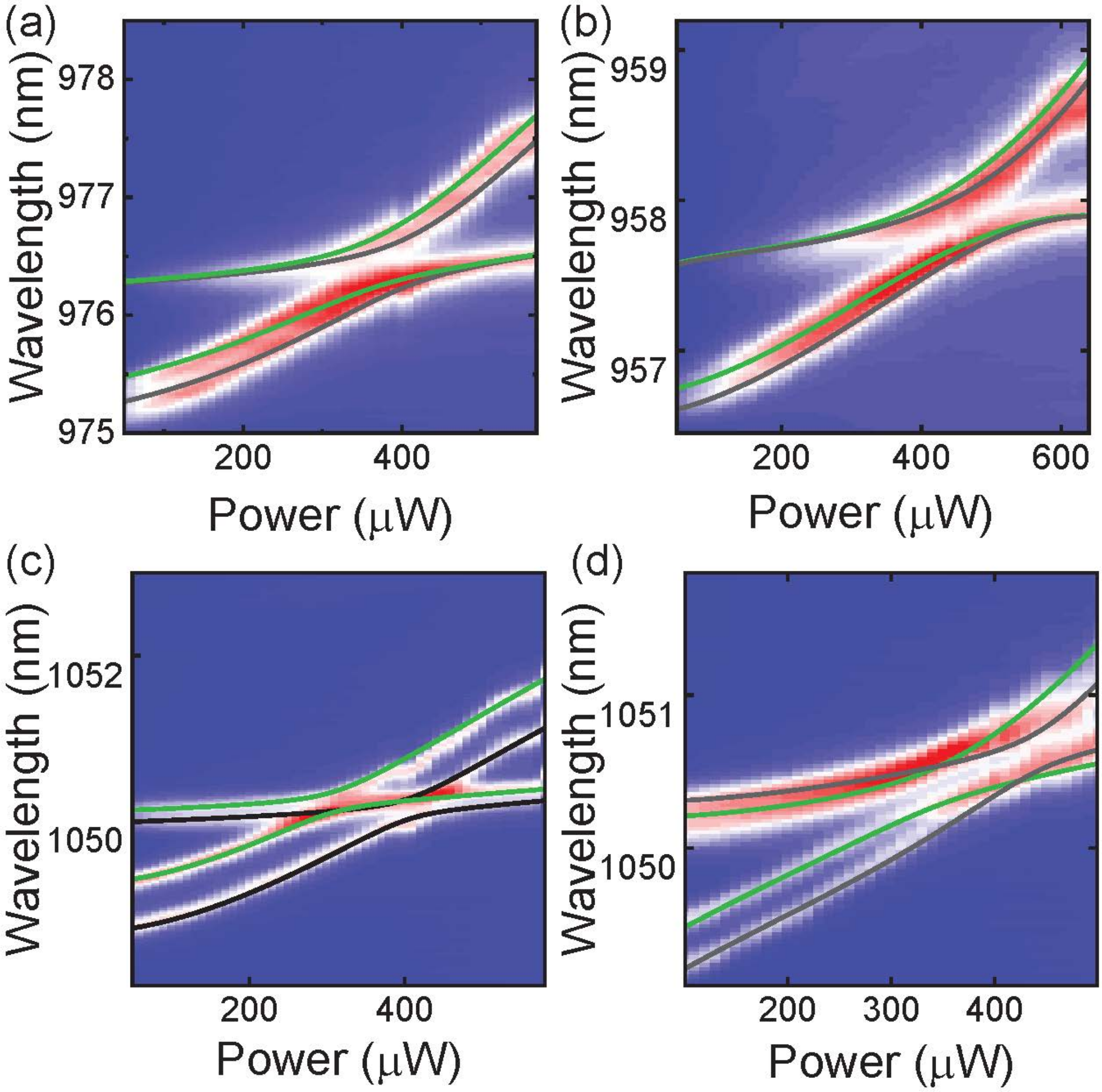

SFig. 1. Excitation-power-dependent PL mappings. All experimental results have two and only two anticrossings. The backscattering coupling strengths are (a)(b) $J_a = 0$ and $J_b \neq 0$, (c) $J_a J_b = g^2$, (d) $J_a J_b < 0$ and $J_a \neq -J_b$.

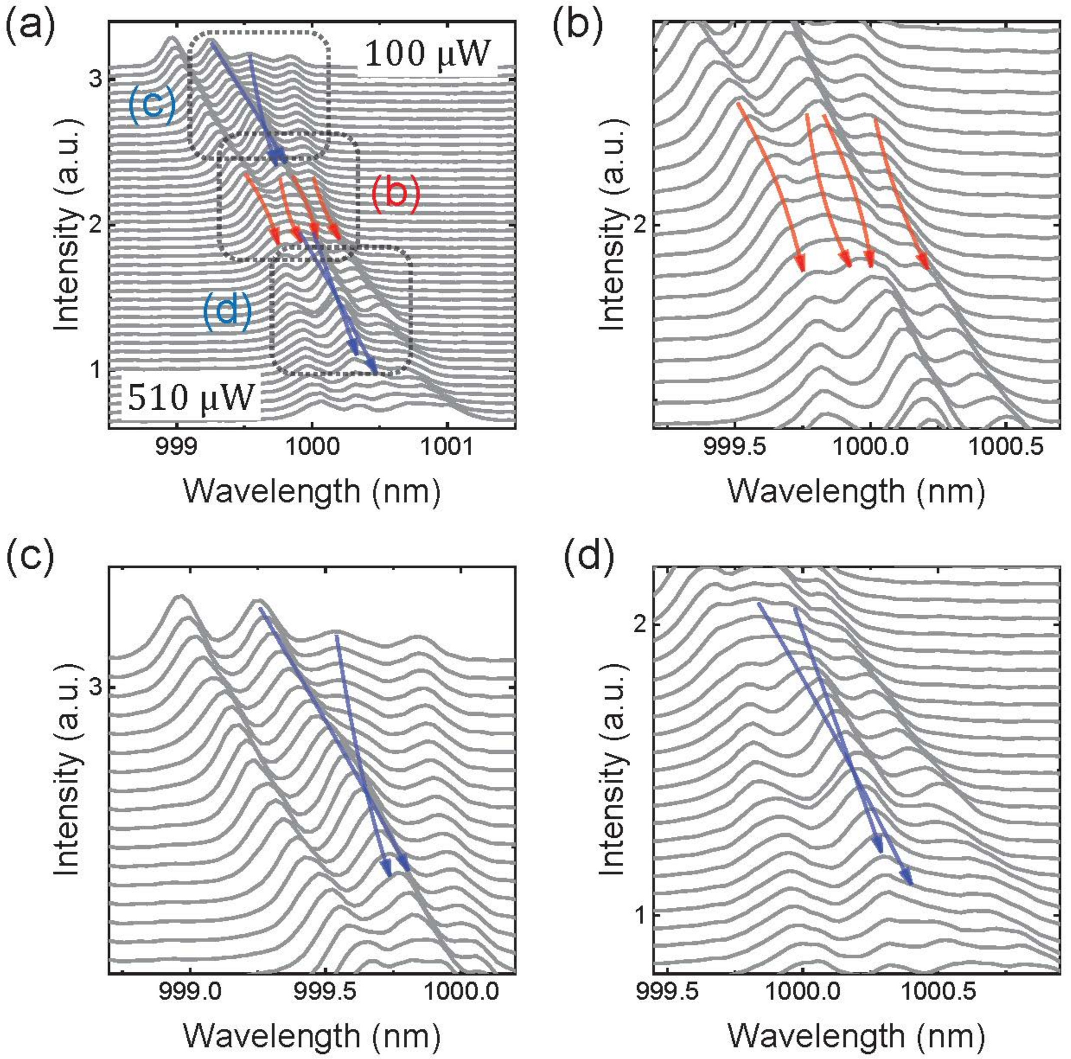

SFig. 2. (a) The PL spectra of Fig. 3(c) in the paper. Red arrows refer to anticrossings. Blue arrows refer to crossings. (b)-(d) Enlarged views of (a) to show the crossings and anticrossings clearly.

C. Eigenspace at Diabolical Points

Our work mainly focuses on the effect of backscattering on the coupled cavities. Thus, it is straightforward to rewrite the Hamiltonian with the basis $\psi_{1,2,3,4}$, which are exactly the eigenstates of two coupled microdisks without backscattering. When two microdisks are at resonance, $\omega_{a,b}$ can be set to zero, then the Hamiltonian is

$$\begin{array}{c} \\ \psi_1 \\ \psi_2 \\ \psi_3 \\ \psi_4 \end{array} \begin{array}{c} \begin{array}{cccc} \psi_1 & \psi_2 & \psi_3 & \psi_4 \end{array} \\ \begin{pmatrix} g & 0 & \frac{J_a+J_b}{2} & \frac{J_a-J_b}{2} \\ 0 & -g & \frac{J_a-J_b}{2} & \frac{J_a+J_b}{2} \\ \frac{J_a+J_b}{2} & \frac{J_a-J_b}{2} & g & 0 \\ \frac{J_a-J_b}{2} & \frac{J_a+J_b}{2} & 0 & -g \end{pmatrix} \end{array}.$$

The effect of two backscattering coupling strengths $J_{a,b}$ on the internal coupling between $\psi_{1,2,3,4}$ can be directly observed in this form. When $J_a = J_b$, the internal coupling only happens between Y-reversal states as ψ_1 and ψ_3, or ψ_2 and ψ_4. In this case, the Hamiltonian is symmetric under both X-reversal operation (exchanging a_{cw} and b_{ccw} as well as a_{ccw} and b_{cw}) and Y-reversal operation. Two anticrossings occur simultaneously at the resonance with eigenvalues $\Omega_{1,3} = g \pm J$ and $\Omega_{2,4} = -g \pm J$. The corresponding eigenstates are $(\psi_1 \pm \psi_3)\,/\,\sqrt{2}$ and $(\psi_2 \pm \psi_4)\,/\,\sqrt{2}$, with odd or even parity in X and Y direction. While when $J_a = -J_b$, the internal couplings between two pairs of reversal states are destructive. The coupling only happens between ψ_1 and ψ_4, or ψ_2 and ψ_3. In this case, the Hamiltonian is only symmetric under Y-reversal operation. The corresponding eigenstates are formed as the superposition of ψ_1 and ψ_4, or ψ_2 and ψ_3, which do not satisfy the Y-reversal symmetry. The eigenstates without Y-reversal symmetry in the system with Y-reversal symmetry indicate a spontaneous symmetry breaking.

Now assume $J_a = -J_b = J$ and $\Omega = \pm\sqrt{g^2 + J^2} = \pm\chi$ with $J = \chi \sin\gamma$ and $g = \chi \cos\gamma$ for brevity. The eigenstates then can be

expressed as

$$S_1 = -\sin\frac{\gamma}{2}\psi_1 + \cos\frac{\gamma}{2}\psi_4\,, S_2 = \cos\frac{\gamma}{2}\psi_1 + \sin\frac{\gamma}{2}\psi_4,$$
$$S_3 = -\cos\frac{\gamma}{2}\psi_2 + \sin\frac{\gamma}{2}\psi_3\,, S_4 = \sin\frac{\gamma}{2}\psi_2 + \cos\frac{\gamma}{2}\psi_3$$

and the eigenvalues are $\Omega_{S_1,S_3} = -\chi$ and $\Omega_{S_2,S_4} = \chi$. Due to the degeneracy, any superposition of the degenerate states, such as S_2 and S_4, is also an eigenstate of the system. The eigenspace constructed with the biorthogonal states can be expressed as

$$S' = \sin\alpha e^{i\beta/2} S_2 + \cos\alpha\, e^{-i\beta/2} S_4$$
$$= \frac{1}{\sqrt{2}}\left(\cos\frac{\beta}{2}\sin\left(\alpha+\frac{\gamma}{2}\right) + i\sin\frac{\beta}{2}\sin\left(\alpha-\frac{\gamma}{2}\right)\right)a_{cw}$$
$$+\frac{1}{\sqrt{2}}\left(\cos\frac{\beta}{2}\cos\left(\alpha-\frac{\gamma}{2}\right) - i\sin\frac{\beta}{2}\cos\left(\alpha+\frac{\gamma}{2}\right)\right)a_{ccw}$$
$$+\frac{1}{\sqrt{2}}\left(\cos\frac{\beta}{2}\sin\left(\alpha-\frac{\gamma}{2}\right) + i\sin\frac{\beta}{2}\sin\left(\alpha+\frac{\gamma}{2}\right)\right)b_{ccw}$$
$$+\frac{1}{\sqrt{2}}\left(\cos\frac{\beta}{2}\cos\left(\alpha+\frac{\gamma}{2}\right) - i\sin\frac{\beta}{2}\cos\left(\alpha-\frac{\gamma}{2}\right)\right)b_{cw},$$

with any α and β.

The eigenstates can also be expressed by the phases θ_a, ϕ_a, θ_b, ϕ_b, φ_1 and φ_2 as mentioned in the paper:

$$S' = \sin\varphi_1 e^{i\varphi_2/2}(\sin\theta_a e^{\frac{i\phi_a}{2}} a_{cw} + \cos\theta_a e^{\frac{-i\phi_a}{2}} a_{ccw})$$
$$+\cos\varphi_1 e^{-i\varphi_2/2}(\sin\theta_b e^{\frac{i\phi_b}{2}} b_{ccw} + \cos\theta_b e^{\frac{-i\phi_b}{2}} b_{cw}).$$

If waveguides are set beside the microdisks, the phases will be embodied by the signals in the waveguides, as schematically shown in SFig. 3. Therefore, the phase control of the microdisk plays an important role in the

directional laser [4]. Additionally, the supermode of two coupled microdisks correlates the two phases together. The correlation indicates the phase shift of signals between the two microdisks or waveguides (bottom in SFig. 3). Such a system with the phase shift can serve as a quantum node which is potentially applicable to the quantum network.

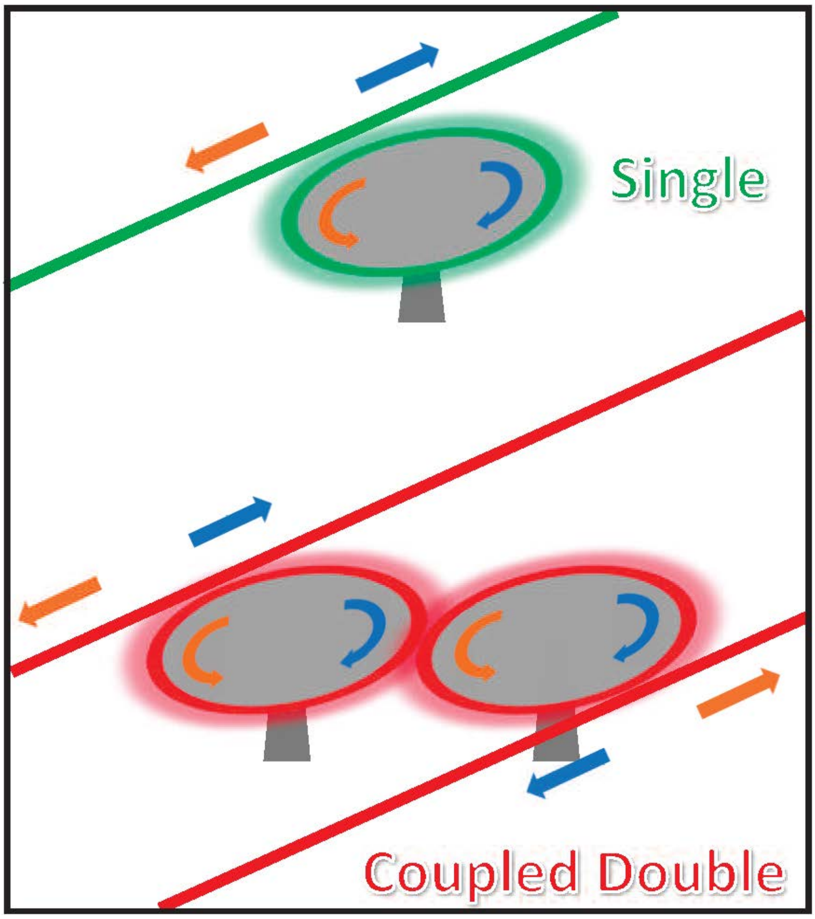

SFig. 3. The schematic for the phases of microdisks embodied by the signals in the waveguides

Combining the Equations above, we can obtain the phases of two microdisks as

$$tan^2 \theta_a = \frac{1 - cos\, 2\alpha\, cos\, \gamma + sin\, 2\alpha\, sin\, \gamma\, cos\, \beta}{1 + cos\, 2\alpha\, cos\, \gamma + sin\, 2\alpha\, sin\, \gamma\, cos\, \beta}$$

$$tan\, \phi_a = \frac{sin\, \beta\, sin\, 2\alpha\, cos\, \gamma}{sin\, \gamma + sin\, 2\alpha\, cos\, \beta}$$

$$tan^2 \theta_b = \frac{1 - cos\, 2\alpha\, cos\, \gamma - sin\, 2\alpha\, sin\, \gamma\, cos\, \beta}{1 + cos\, 2\alpha\, cos\, \gamma - sin\, 2\alpha\, sin\, \gamma\, cos\, \beta}$$

$$tan\, \phi_b = \frac{sin\, \beta\, sin\, 2\alpha\, cos\, \gamma}{- sin\, \gamma + sin\, 2\alpha\, cos\, \beta}$$

These results show the correlation between the phase of two microdisks in the eigenspace, namely, the phase of microdisk B is the function of the phase of microdisk A. The result

$$\theta_b = arctan \left(\frac{tan\, \theta_a - sin\, \gamma}{1 - tan\, \theta_a\, sin\, \gamma} \right)$$

in the paper is the special case with $\beta = \phi_a = \phi_b = 0$. Similarly, for the construction of S_1 and S_3, we can get

$$\theta_b = arctan \left(\frac{tan\, \theta_a + sin\, \gamma}{1 + tan\, \theta_a\, sin\, \gamma} \right).$$

These two results correspond to the solid and dashed redlines in Fig. 2(b) in the paper.

This non-linear correlation is the significant difference compared to coupled microdisks without backscattering, as shown in Fig. 2(b) in the paper. Without backscattering, the phases of two microdisks are the same (the blue line in Fig. 2). Thereby, if phase $\theta_a$is input in microdisk A, the output in microdisk B will have the same phase $\theta_b = \theta_a$ In contrast, with backscattering, two phases are different (red line in Fig.2). The phase

difference between input and output could be used for the phase shift of signals in the quantum nodes as shown in SFig. 3. Meanwhile, with high excitation power the cavity-dot system can also be used for lasers. The output laser in the waveguide beside microdisk B (θ_b) could be controlled by θ_a or γ (gap between microdisks), indicating potential applications such as controllable directional lasers.

II. FABRICATION AND MEASUREMENT:

A. Material and Sample Fabrication

The material of our device was grown by molecular beam epitaxy which consists of a 250-nm-thick GaAs slab on a 1-μm-thick AlGaAs sacrificial layer. One layer of InAs quantum dots as the embedded scatterers and light sources with a density of about 30 μm^{-2} was grown in the middle of the GaAs slab. SFigure 4(a) shows the atomic force microscope (AFM) image of the ensemble QDs and SFig. 4(b) shows the corresponding wide emission wavelength range. The fabrication process involves first patterning of the masks for the subsequent etching by electron beam lithography, followed by the dry etching using inductively coupled plasma to form circular pillars under the protection of the patterning masks, and then wet etching using HF solutions was performed to dissolve the sacrificial layer and form a supporting pedestal under each microdisk.

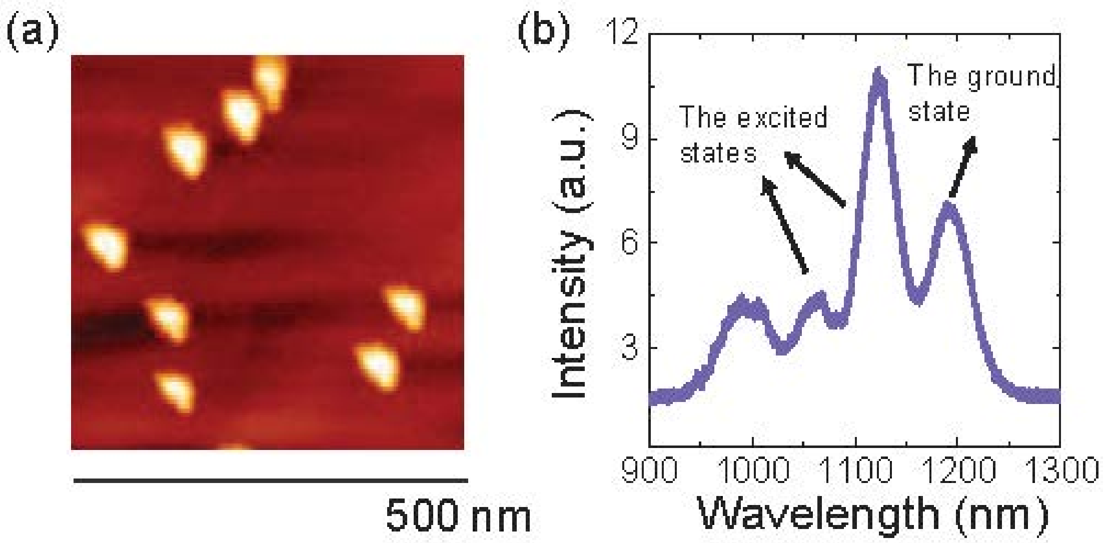

SFig. 4. (a) AFM image of the ensemble of quantum dots on a device wafer. (b) PL spectrum of the ensemble of quantum dots with one ground state and two excited states.

As mentioned in the paper, the competition between two types of scatterers is related to the area/perimeter ratio namely the microdisk radius r. Thus, a proper microdisk size is the key for the balance between two different types of scatterers. Previous work has reported on the strongly variable mode splitting $2|J|$ with a proper microdisk radius of 10 μm [5], which is explained in this paper by the balance between negative and positive distribution to J. In this previous work [5], the QD radius is 10 nm and QD density is 50 μm^{-2}. Compared to the previous work, our QD size nearly quadruples ($\times$ 4 size, $\times 4^2$ volume), and the density nearly halves (30/50) as shown in SFig. 4(a). Thus, for the balance between defects and QDs, the microdisk radius is designed to be $10 \times (1/4)^2 \times (50\ /\ 30) \approx 1\ \mu m$ in both single microdisks and two coupled microdisks. In two coupled microdisks, the gap between them is designed from 50 to 130 nm to achieve a variable g.

B. Measurement Methods

To investigate optical properties at a low temperature, the device was mounted on a 3-dimensional nano-positioner and cooled down to approximately 4.2 K by exchanging helium gas with a helium bath. A conventional confocal micro-photoluminescence (PL) system was used for the collection of the PL spectra and the excitation of the device. The PL spectra were collected by a linear array of InGaAs detectors dispersed through a spectrometer. The resolution of the spectrometer is 0.1 nm. QDs are the main active material to excite the cavity modes through the non-resonant excitation by a solid-state laser with the emission wavelength of 532 nm. To simplify, firstly GaAs substrate is excited by the laser, subsequently the wet layer in the middle of GaAs substrate is excited, then the QDs are excited and finally the cavity modes are excited by emission from several states of the QDs ensemble in a continuous and wide spectrum. Besides, the emission direction of QD is random, and QD can also emit in two polarizations. Thus, QDs will not selectively excite cavity modes with a preferential propagation direction or polarization. Therefore, as an ensemble, the multiple QDs can excite all cavity modes within the spectral range of emission and the effect of emissions of QDs on the properties of cavity can be omitted here. Selective excitation and heating were used to control the detuning between ω_a and ω_b [3].

As the cavity is selectively heated, the mode red shifts due to the thermo-

optic effect: excitation power increases the temperature, thereby, increases the refractive index. If microdisk A is selectively excited, the thermo-optic effect resulting in the redshift can be expressed by $\omega_a = \omega' + B / \left(1 + e^{(P-P_0)/C}\right)$ [3]. ω' is a coefficient related to the detuning from the resonance frequency before increasing the excitation power. B is a constant with a value around 1800. C is around 300 μW. P_0 is approximately 600 μW, P is the laser excitation power at the microdisks. The redshift of the other microdisk B can be expressed by $\omega_b = \omega'' + F * P + G * P^2$, where ω'' is related to the detuning to the resonance frequency. $F \approx 0.01$ and $G \approx 0.000002$. These results refer to the perfect selective excitation, which means one microdisk is uniformly excited while the other microdisk is not excited at all. The perfect selective excitation is hard to achieve in the experiment. As a result, in the imperfect selective excitation, parameters (B, C, F, G and P_0) related to the shift rates will change slightly for different modes or cavities. Additionally, the equations above are mainly available with low excitation power. When the excitation power is very high, the absorption of the excitation laser may be saturate. Thus, the temperature will increase relative slowly with high excitation power.

SFigure 5 shows the PL spectrum of the cavity modes in a single microdisk (a), and in double microdisks (b). All microdisks were designed with the same radius of 1 μm. Thus, modes in each cavity are similar. For

most peaks in SFig. 5(a), the corresponding peaks can be found

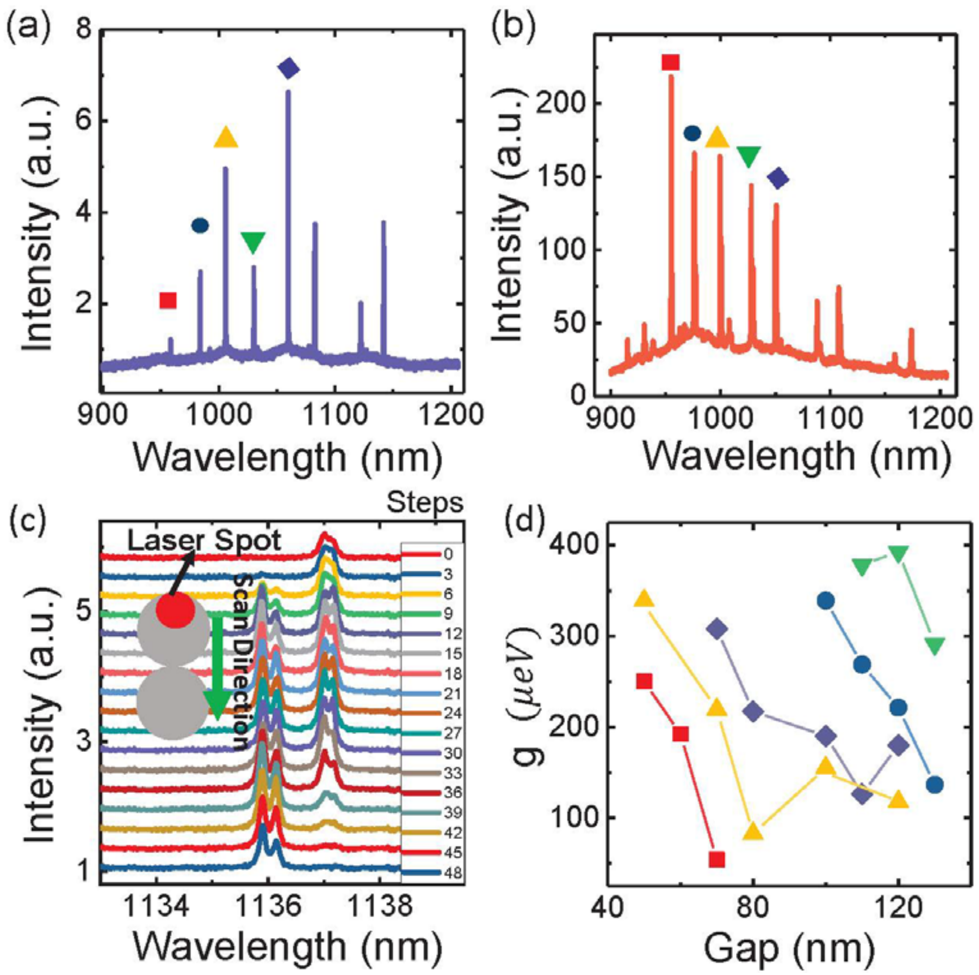

SFig. 5. (a) PL spectrum of a single microdisk. (b) PL spectrum of two coupled microdisks. Symbols of the same color in (a) and (b) refer to the same mode. (c) Excitation-position-dependent PL spectra of two coupled microdisks. The intensity variation indicates the origin of the mode. (d) The decrease of g with the gap. Different modes have different decrease rates. Symbols of the same color represent the same mode in (a) and (b).

in SFig. 5(b) with similar wavelengths, as marked by the color symbols. While fabrication imperfection can lead to difference between microdisks. As a result, modes in different cavities are similar but not identical. In SFig. 5(a), each mode refers to two split peaks, while in SFig. 5(b) each mode

refers to four split peaks originates from two coupled microdisks, as clearly shown in SFig. 5(c). SFigure 5(c) shows the excitation-position-dependent PL spectra. The excitation laser spot (large red dot) is adjusted from one microdisk to another microdisk by moving the sample stage, as marked by the green arrow. This method is used to identify the origin of modes. For example, in SFig. 5(c), when the excitation position is in the upper microdisk, the intensities of two peaks with longer wavelengths are stronger; when the excitation position is in the bottom microdisk, the intensities of two peaks with shorter wavelengths are stronger. Thus, we can identify that two peaks with longer wavelengths are from the upper microdisk, and two peaks with shorter wavelengths are from the bottom microdisk. Due to that the thermo-optic effect can only redshift the cavity modes, we need to adjust the excitation laser spot to the microdisk with shorter cavity mode wavelengths. Then the excitation power is increased to heat the microdisk to adjust microdisks into resonance.

The coupling strength between two microdisks g is extracted by fitting the experimental data with the theoretical analysis in Sec. IA. SFigure 5(d) shows the values of g in the systems with different gaps. The dots with the same color refer to the same mode in different coupled cavities. The decrease of g with the gap between two microdisks can be clearly observed, paving the way to control the phase shift by controlling the gap (γ) as mentioned in the paper. Besides, it can be observed that the

decreases of g are different for different modes. This difference is attributed to the different mode field distribution, which affects the energy exchange between two microdisks. The whispering gallery mode with a shorter wavelength usually has less evanescent wave leakage into the gap, resulting in a smaller g and a faster decrease with the gap [6].

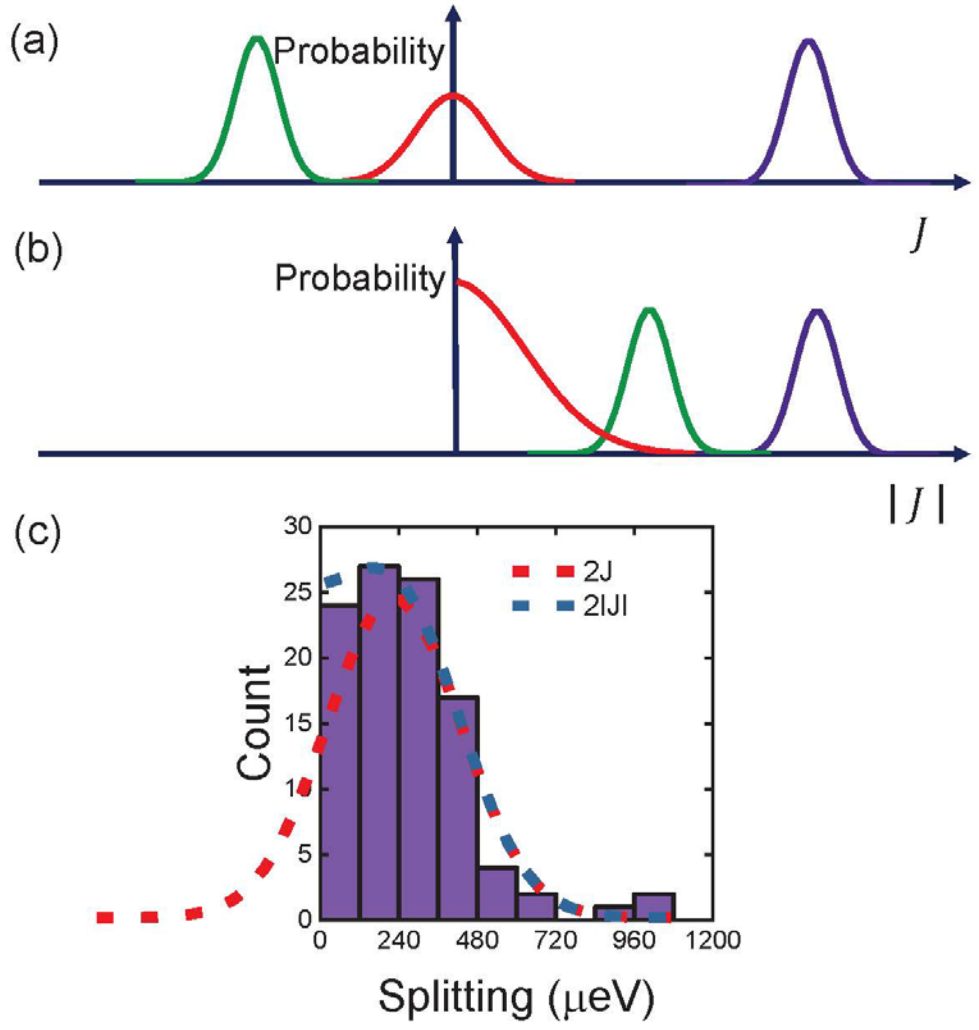

SFig. 6. (a) The schematic of J distribution with different competition between two types of scatterers. (b) The corresponding distribution of the splitting $2|J|$. (c) The statistics of splitting extracted from the experimental data.

III. BALANCED COMPETITION:

In our work, due to the random positions of QDs and defects, the backscattering coupling strength J is strongly variable with different modes. The distribution of J is determined by the competition between two types of scatterers: QDs and defects, making negative or positive contributions to the backscattering coupling strength J. The schematic of the competition is shown in SFig. 6(a). When the microdisk size is very small, defects dominate and lead to negative J (green line). When the microdisk size is very large, QDs dominate and lead to positive J (purple line). In these two cases, approximately only one type of scatterers dominate. Thus, the mode splitting $2|J|$ will has a large average value with the distribution away from zero (green and purple lines in SFig. 6(b)), which has been reported previously [7]. In contrast, the competition between QDs and defects is close to balance in our work due to the proper microdisk size. Therefore, the distribution of J is from negative to positive (red line in SFig. 6(a)), and the corresponding mode splitting $2|J|$ will appear like a half Gaussian distribution (red line in SFig. 6(b)). The half Gaussian distribution agrees well with the experimental data as shown in SFig. 6(c). The good agreement between the theoretical analysis and the experimental result demonstrates the balance of the competition in our work, which provides the basis for the DPs with $J_a = -J_b$.

References:

[1] T. J. Kippenberg, S. M. Spillane, and K. J. Vahala, Modal coupling in traveling-wave resonators, Opt. Lett. 27, 1669 (2002).

[2] C. Schmidt, A. Chipouline, T. Käsebier, E.-B. Kley, A. Tünnermann, T. Pertsch, V. Shuvayev, and L. I. Deych, Observation of optical coupling in microdisk resonators, Phys. Rev. A 80, 043841 (2009).

[3] M. Benyoucef, S. Kiravittaya, Y. F. Mei, A. Rastelli, and O. G. Schmidt, Strongly coupled semiconductor microcavities: A route to couple artificial atoms over micrometric distances, Phys. Rev. B 77, 035108 (2008).

[4] S. Longhi and L. Feng, Unidirectional lasing in semiconductor microring lasers at an exceptional point, Photon. Res. 5, B1 (2017).

[5] B. D. Jones, M. Oxborrow, V. N. Astratov, M. Hopkinson, A. Tahraoui, M. S. Skolnick, and A. M. Fox, Splitting and lasing of whispering gallery modes in quantum dot micropillars, Opt. Express 18, 22578 (2010).

[6] H. Lin, J.-H. Chen, S.-S. Chao, M.-C. Lo, S.-D. Lin, and W.-H. Chang, Strong coupling of different cavity modes in photonic molecules formed by two adjacent microdisk microcavities, Opt. Express 18, 23948 (2010).

[7] L. He, Şahin Kaya Özdemir, J. Zhu, F. Monifi, H. Yımaz, and L. Yang, Statistics of multiple-scatterer-induced frequency splitting in whispering gallery microresonators and microlasers, New J. Phys. 15, 073030 (2013).
